# Agonists and knockdown of estrogen receptor β differentially affect invasion of triple-negative breast cancer cells in vitro

**DOI:** 10.1186/s12885-016-2973-y

**Published:** 2016-12-21

**Authors:** Susanne Schüler-Toprak, Julia Häring, Elisabeth C. Inwald, Christoph Moehle, Olaf Ortmann, Oliver Treeck

**Affiliations:** 1 Department of Gynaecology and Obstetrics, University Medical Center Regensburg, Caritas-Hospital St. Josef, Landshuter Str. 65, 93053 Regensburg, Germany; 2 Center of Excellence for Fluorescent Bioanalytics (KFB), Am BioPark 9, 93053 Regensburg, Germany

**Keywords:** Estrogen receptor beta, Triple-negative breast cancer, Cell culture, Invasion

## Abstract

**Background:**

Estrogen receptor β (ERβ) is expressed in the majority of invasive breast cancer cases, irrespective of their subtype, including triple-negative breast cancer (TNBC). Thus, ERβ might be a potential target for therapy of this challenging cancer type. In this in vitro study, we examined the role of ERβ in invasion of two triple-negative breast cancer cell lines.

**Methods:**

MDA-MB-231 and HS578T breast cancer cells were treated with the specific ERβ agonists ERB-041, WAY200070, Liquiritigenin and 3β-Adiol. Knockdown of ERβ expression was performed by means of siRNA transfection. Effects on cellular invasion were assessed in vitro by means of a modified Boyden chamber assay. Transcriptome analyses were performed using Affymetrix Human Gene 1.0 ST microarrays. Pathway and gene network analyses were performed by means of Genomatix and Ingenuity Pathway Analysis software.

**Results:**

Invasiveness of MBA-MB-231 and HS578T breast cancer cells decreased after treatment with ERβ agonists ERB-041 and WAY200070. Agonists Liquiritigenin and 3β-Adiol only reduced invasion of MDA-MB-231 cells. Knockdown of ERβ expression increased invasiveness of MDA-MB-231 cells about 3-fold. Transcriptome and pathway analyses revealed that ERβ knockdown led to activation of TGFβ signalling and induced expression of a network of genes with functions in extracellular matrix, tumor cell invasion and vitamin D3 metabolism.

**Conclusions:**

Our data suggest that ERβ suppresses invasiveness of triple-negative breast cancer cells in vitro. Whether ERβ agonists might be useful drugs in the treatment of triple-negative breast cancer, has to be evaluated in further animal and clinical studies.

**Electronic supplementary material:**

The online version of this article (doi:10.1186/s12885-016-2973-y) contains supplementary material, which is available to authorized users.

## Background

Ten to twenty percent of all breast cancers are triple-negative breast cancers (TNBC) [1]. This breast cancer subgroup lacks expression of estrogen receptor alpha (ERα) and progesterone receptor (PR) as well as human epidermal growth factor receptor 2 (HER2) amplification. TNBCs are more frequent in younger patients and tumors are generally larger in size. Moreover, TNBCs are more aggressive, of higher grade and often have lymph node involvement at diagnosis [1, 2]. As patients with TNBC do not benefit from targeted therapies with tamoxifen or trastuzumab [3–5], they have a poorer prognosis and a higher rate of distant recurrence than women with other breast cancer subtypes [2, 6]. Less than one third of women with metastatic TNBC survive 5 years, and almost all die of their disease despite adjuvant chemotherapy [6]. Most of TNBCs can be classified as basal-like either by immunohistochemistry or by correlation to the intrinsic molecular breast cancer subtypes [7–9]. Basal-like tumors express markers of the myoepithelium of the normal mammary gland, like epidermal growth factor receptor (EGFR), p63 and the basal cytokeratins CK14, CK5/6 and CK17 [10, 11].

In contrast to estrogen receptor α (ERα), the second estrogen receptor, ERβ has been shown to be expressed in all molecular subtypes of breast cancer, including 60% of basal-like tumors [12]. Thus, ERβ could be an interesting therapy target for patients with TNBC. ERβ has been suggested to act as a tumor-suppressor in breast tissue, because its expression declines during carcinogenesis, its knockdown increased proliferation of mammary epithelial and breast cancer cells, whereas its overexpression inhibited tumor cell proliferation [13–17]. Previously, ERβ status has been reported to affect clinical outcome of TNBC [18]. However, the role of ERβ in regulation of breast cancer cell invasiveness is only beginning to be understood. Previously, ERβ has been reported to enhance adhesion of ERα-positive breast cancer cells by increase of integrin expression [19]. A recent study reported that ERβ was able to repress epithelial to mesenchymal transition and invasion of basal-like breast cancer cells by destabilizing EGFR [20].

In this study, we further approached the role of ERβ in invasiveness of TNBC cells. We knocked down ERβ in TNBC cells and performed transcriptome and gene network analyses to elucidate, whether genes with functions in tumor cell invasion would be regulated. Additionally, we examined whether treatment with ERβ agonists would affect invasiveness of TNBC cell lines in vitro.

## Methods

### Material

Phenol red-free DMEM culture medium was obtained from Invitrogen (Karlsruhe, Germany), FCS was purchased from PAA (Pasching, Austria). MDA-MB-231 and HS578T breast cancer cells were obtained from American Type Culture Collection (Manassas, USA). RNeasy Mini Kit was obtained from Qiagen (Hilden, Germany). Transfectin reagent was obtained from BioRad (Hercules, USA). OptiMEM medium were purchased at Invitrogen (Karlsruhe, Germany). ESR2 and control siRNAs were from Ambion (Life Technologies, USA). Serum Replacement 2 (SR2) cell culture supplement was from Sigma-Aldrich (Deisenhofen, Germany). ERβ agonists ERB-041 and WAY-200070 were from Tocris (Bristol, UK). 5α-androstane-3β, 17β-diol (3β-Adiol) was from Sigma (Deisenhofen, Germany) and Liquiritigenin from Extrasynthese (Lyon, France).

### Cell culture, transfection and proliferation assays

MDA-MB-231 and HS578T cells were maintained in DMEM/F12 medium supplemented with 10% FCS. Cells were cultured with 5% CO_2_ at 37 °C in a humidified incubator. For transfection, 4 × 10^5^ cells per well of a 6-well dish were seeded in DMEM/F12 containing 10% FCS. The next day, 2 ml fresh culture medium was added to the cells, transfection solution was prepared in OptiMEM medium (Invitrogen) using 5 μl Transfectin reagent (BioRad) and a mix of three ESR2 siRNAs (10 nM each) (or 10 nM of siRNA specific for CYP24A1, CXCL14 or negative control siRNA) and was added to the cultured cells. The siRNA mix contained three different ESR2-specific Silencer siRNAs (siRNA IDs 145909, 145910, 145911, Ambion), targeting exons 1, 2 and 3 of ESR2 mRNA. For knockdown of CYP24A1 and CXCL14, further Silencer siRNAs were used (siRNA IDs 106233 and 137806, respectively, Ambion). As a negative control, Silencer Negative control siRNA #1 (Ambion) was used. Gene knockdown of ESR2, CYP24A1 and CXCL14 was verified by means of Western blot analysis 72 h after siRNA treatment as described below. For cell proliferation assays, cells cultured in DMEM/F12 supplemented with 10% FBS were seeded in 96-well plates in triplicates (1000 cell/well). On days 0, 2, 3 and 4 relative numbers of viable cells were measured using the fluorimetric, resazurin-based Cell Titer Blue assay (Promega) according to the manufacturer’s instructions at 560Ex/590Em nm in a Victor3 multilabel counter (PerkinElmer, Germany). Cell growth was expressed as percentage of day 0. Growth data were statistically analyzed by the Kruskal–Wallis one-way analysis of variance.

### Invasion assays

Tumor cell invasion was measured by assessment of breast cancer cell invasion through an artificial basement membrane using the 24-well Cultrex BME cell invasion assay (Trevigen, USA), a modified Boyden-chamber transwell assay with 8 μm pore size, according to the manufacturer’s instructions. BME (basement membrane extract) is a soluble form of basement membrane purified from Engelbreth-Holm-Swarm (EHS) tumor, mainly consisting of laminin, collagen IV, entactin, and heparin sulfate proteoglycan. Briefly, 100 μl ice-cold liquid BME extract (10 mg/ml) was placed on top of the insert membranes and polymerized at 37 °C over night to form a reconstituted basement membrane gel of about 3 mm thickness. 50000 MDA-MB-231 or HS578T cells (plus/minus ERβ agonists, calcitriol or CXCL14 chemokine) or the same number of cells previously transfected with siRNA specific for ESR2, CYP24A1 or CXCL14, serum starved in SR2 medium, were seeded the day after treatment (or 2 days after treatment with the ERβ agonists) on top of the BME coated inserts. The lower compartment was filled with 600 μl of DMEM-F12 supplemented with 10% FCS as a chemoattractant. After 48 h of invasion in a humidified incubator with 5% CO_2_ at 37 °C, relative numbers of cells invaded into the bottom chamber were relatively quantified using the fluorimetric Cell Titer Blue assay (Promega) as described above. As negative controls, samples without chemoattractant were measured. Cell proliferation used for calculation of the corrected invasion rate was determined in parallel experiments using the same assay.

### RNA preparation and real-time RT-PCR

Total RNA was isolated from 30 to 80 mg frozen tissue or from cell lines (10^6^ cells) by means of Trizol reagent (Invitrogen, Karlsruhe, Germany) according to manufacturer’s protocol. RNA purity and concentration was analyzed by spectrophotometry. From each sample, 500 ng of total RNA was reverse transcribed to cDNA using 40 units of M-MLV Reverse Transcriptase and RNasin (Promega, Mannheim, Germany) with 80 ng/μl random hexamer primers (Invitrogen, Karlsruhe, Germany) and 10 mM dNTP mixture (Fermentas, St. Leon-Rot, Germany) according to the manufacturer’s instructions. After reverse transcription, specific transcript levels were determined by real-time PCR. For this purpose, 4 μl of cDNA were amplified using LightCycler® FastStart DNA Master^PLUS^ SYBR Green I (Roche Diagnostics GmbH, Mannheim, Germany) and 5 mM of each primer (Additional file 1: File S1). Oligonucleotides (Metabion, Planegg-Martinsried, Germany) were designed intron-spanning to avoid genomic contaminations.

Real-time PCRs were carried out in a LightCycler® 2.0 Instrument (Roche, Mannheim, Germany) under the following conditions: initial denaturation at 95 °C for 15 min, followed by 45 cycles with 10 s denaturation at 95 °C, 5 s annealing at 60 °C and 12 s extension at 72 °C. The PCR program was completed by a standard melting curve analysis. Negative controls were prepared by adding distilled water instead of cDNA. To verify the identity of the PCR products, they were initially analyzed by electrophoresis in 1.5% agarose gels and stained with ethidium bromide. After size check, each PCR product was then purified using the “QIAquick Gel Extraction Kit” (Qiagen, Hilden, Germany), following the manufacturer’s protocol and verified by sequencing (Eurofins MWG Operon, Ebersberg, Germany). In all RT-PCR experiments, a 190 bp β-actin fragment was amplified as reference gene using intron-spanning primers actin-2573 and actin-2876. Data from two independent PCR experiments per sample were analyzed using the comparative ΔΔC_T_ method [21] calculating the difference between the threshold cycle (C_T_) values of the target and reference gene of each sample and then comparing the resulting Δ C_T_ values between different samples.

### Western blot analysis

Seventy-two hours after transfection, MDA-MB-231 were lysed in RIPA buffer (1% (*v/v*) Igepal CA-630, 0.5% (*w/v*) sodium deoxycholate, 0.1% (*w/v*) sodium dodecyl sulphate (SDS) in phosphate-buffered solution (PBS) containing aprotonin and sodium orthovanadate. Aliquots containing 10 μg of protein were resolved by 10% (*w/v*) SDS–polyacrylamide gel electrophoresis, followed by electrotransfer to a PVDF hybond (Amersham, UK) membrane. Immunodetection was carried out using monoclonal ESR2 antibody 14C8 (1:500), (ab288, Abcam, Germany), CYP24A1 polyclonal antibody (ab175976, Abcam, Germany) diluted 1:300 in PBS containing 5% skim milk (*w/v*), polyclonal CXCL14 antibody (1:250) (ab36622, Abcam, Germany), monoclonal tenascin-c antibody [EPR4219] (1:500) (ab108930, Abcam, Germany), polyclonal MMP13 antibody (1:1000) (ab39012, Abcam) and β-actin antibody (1:500) (ab8226, Abcam) followed by horseradish peroxidase conjugated secondary antibody (1:20000) which was detected using chemiluminescence (ECL) system (Amersham, Buckinghamshire, UK). The Western blot results from three independent protein isolations were densitometrically analyzed (ImageJ, NIH) and expressed in percentage of cell transfected with negative control siRNA.

### GeneChip^TM^ microarray assay

Processing of four RNA samples (two biological replicates from MDA-MB-231 cells transfected with ESR2 siRNAs or control siRNA as described above) was performed at the local Affymetrix Service Provider and Genomics Core Facility, “KFB - Centre of Excellence for Fluorescent Bioanalytics” (Regensburg, Germany; www.kfb-regensburg.de). Sample preparation for microarray hybridization was carried out as described in the Affymetrix GeneChip® Whole Transcript (WT) Sense Target Labelling Assay manual. 300 ng of total RNA were used to generate double-stranded cDNA. Subsequently synthesized cRNA (WT cDNA Synthesis and Amplification Kit, Affymetrix) was purified and reverse transcribed into single-stranded (ss) DNA. After purification, the ssDNA was fragmented using a combination of uracil DNA glycosylase (UDG) and apurinic/apyrimidinic endonuclease 1 (APE 1). Fragmented DNA was labelled with biotin (WT Terminal Labelling Kit, Affymetrix), and 2.3 μg DNA were hybridized to the GeneChip Human Gene 1.0 ST Array (Affymetrix) for 16 h at 45 °C in a rotating chamber. Hybridized arrays were washed and stained in an Affymetrix Washing Station FS450 using preformulated solutions (Hyb, Wash & Stain Kit, Affymetrix), and the fluorescent signals were measured with an Affymetrix GeneChip® Scanner 3000-7G.

### Microarray data analysis

Summarized probe signals were created by using the RMA algorithm in the Affymetrix GeneChip Expression Console Software and exported into Microsoft Excel. Data was then analysed using Ingenuity IPA Software (Ingenuity Systems, Stanford, USA) and Genomatix Pathway Analysis software (Genomatix, Munich, Germany). Genes with more than 2-fold changed mRNA levels after ERβ knockdown in both biological replicates were considered to be differentially expressed and were included in the analyses.

## Results

### Characterization of the employed breast cancer cell lines

First we tested receptor expression of MDA-MB-231 and HS578T cells to characterize the cell culture models employed in this study. For comparison we included MCF-7 cells, known to express ERs and PR and also SK-BR3 cells, which overexpress HER2. MDA-MB-231 and HS578T cells did only express extremely low or even undetectable mRNA levels of ERα, PR or HER2, as expected from triple-negative breast cancer cells. In contrast, they strongly expressed EGFR mRNA. ERβ transcript levels were higher in MDA-MB-231 cells than in MCF-7 and HS578T cells (Additional file 2: Figure S2).

### Effect of ERβ agonists on invasion of MDA-MB-231 and HS578T cells

**Fig. 1:**
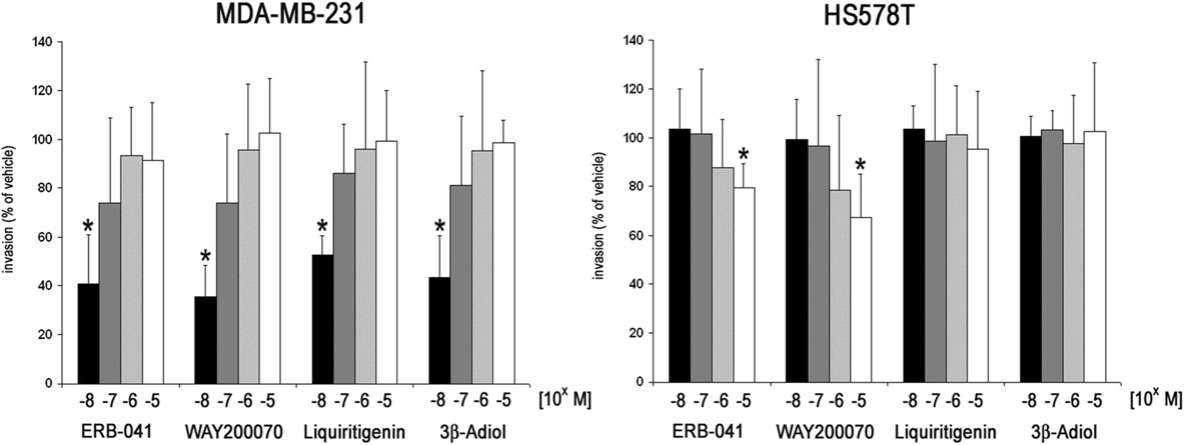
Effect of different ERβ agonists on invasion of triple negative MDA-MB-231 and HS578T breast cancer cells. Cellular invasion through a 3 mm gel of reconstituted basement membrane was determined using a modified Boyden chamber. Cells were pre-treated for 48 h with the indicated concentrations of ERβ agonists, seeded on top of the basement membrane gel in the presence of the same agonist concentrations, and invasion was determined after further 48 h as described in the Materials and Methods section. Values are expressed in percent of invasion of vehicle-treated cells. *p < 0.05 vs vehicle control. (*n* = 4). (Kruskal-Wallis H-test with Bonferroni post-hoc test)

The employed cell lines MDA-MB-231 and HS578T had a comparable invasion capacity (Additional file 3: Figure S3). To examine the role of ERβ in invasion of TNBC cells, we first treated both cell lines with a panel of four ERβ agonists. Treatment with all ERβ agonist decreased invasion of MDA-MB-231 cells and, to a lesser extent, of HS578T cells. While we tested agonist concentrations from 10 nM to 10 μM, only treatment with 10 nM of ERβ agonists had a statistically significant effect on invasion of MDA-MB-231 cells. Ten nanometre of ERB-041 decreased invasion down to 39.8% (*p* < 0.05), 10 nM of WAY200070 reduced invasion down to 37.1% (*p* < 0.05), 10 nM of 3β-Adiol down to 42.8% (*p* < 0.05) and the same concentration of Liquiritigenin decreased invasion down to 53.5% (*p* < 0.05). In contrast, invasiveness of HS578T cells expressing lower levels of ERβ was only inhibited by the highest concentration of ERB-041 and WAY-200070 (10 μM), but was not affected by the other two agonists (Fig. 1). None of the ERβ agonists tested did affect proliferation of these cell lines in a significant manner (data not shown).

### Effect of an ERβ knockdown on invasiveness of breast cancer cells

**Fig. 2:**
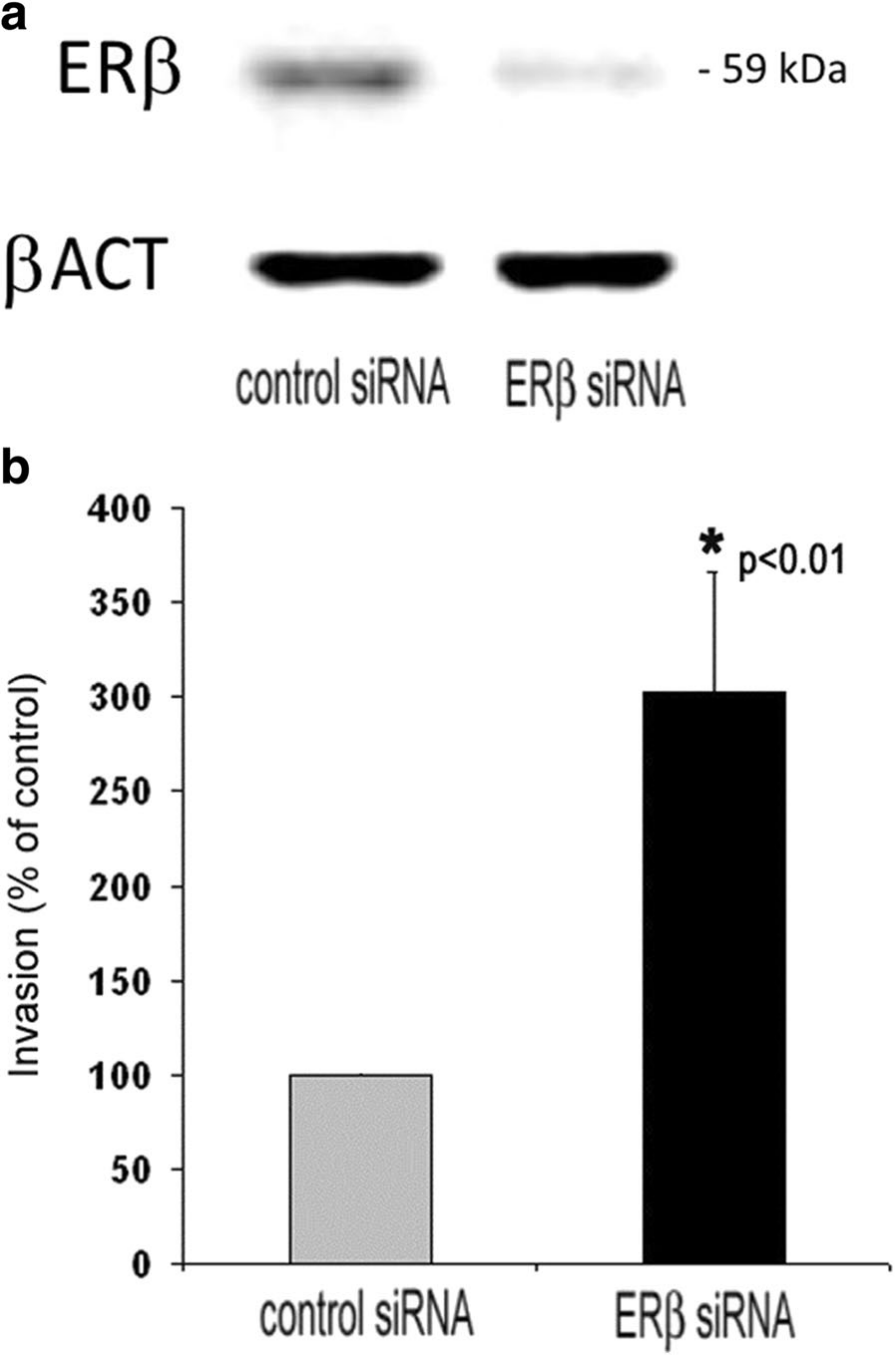
Effect of ERβ knockdown on invasion of MDA-MB-231 cells. **a** Effect of treatment with ESR2 siRNA for 72 h on ERβ protein expression in MDA-MB-231 cells as assessed by Western blot analysis. **b** Effect of ERβ knockdown on cellular invasion of MDA-MB-231 cells through a basement membrane in vitro. The day after transfection, cells were seeded on top of a 3 mm reconstituted basement membrane gel, and invasion was determined after further 48 h as described in the Materials and Methods section. Values are expressed in percentage of invasion of control-transfected cells. *vs. negative control siRNAs (*n* = 3) (unpaired t‑test, two‑tailed)

We now wanted to find out whether knockdown of ERβ would in turn be able to induce breast cancer cell invasion. Given that MDA-MB-231 cells turned out to be much more sensitive to ERβ agonists, and had a higher expression of this receptor, we chose this cell line for knockdown of ERβ. Seventy-two hours after transfection with ERβ-specific siRNAs, a maximum suppression of total ERβ transcript levels down to 11.8% was observed (data not shown). Western blot analysis confirmed knockdown of ERβ protein expression after 72 h of transfection (Fig. 2a). Using these ERβ knockdown cells together with cells transfected with negative control siRNA, we performed further in vitro invasion assays to examine the action of this gene in TNBC cell invasion. To be sure that ERβ expression was significantly reduced during the whole invasion assay, MDA-MB-231 cells were seeded onto the basement membrane 24 h after siRNA transfection, and invasion was measured after further 48 h. MDA-MB-231 cells transfected with ERβ siRNA showed an about 3-fold higher invasion level than cells treated with negative control siRNA (Fig. 2b). With regard to cell proliferation, only knockdown of ERβ in MDA-MB-231 cells, but not in HS578T cells significantly accelerated proliferation (Additional file 4: Figure S4).

### Effect of an ERβ knockdown on transcriptome of MDA-MB-231 cells

To elucidate the molecular mechanisms underlying the effect of this receptor on TNBC invasion, we examined the effect of an ERβ knockdown on transcriptome of MDA-MB-231 cells. For this purpose, we compared the transcriptome of these knockdown cells with MDA-MB-231 cells transfected with negative control siRNA by DNA microarray analysis (Affymetrix Human Gene 1.0 ST Arrays).

**Fig. 3:**
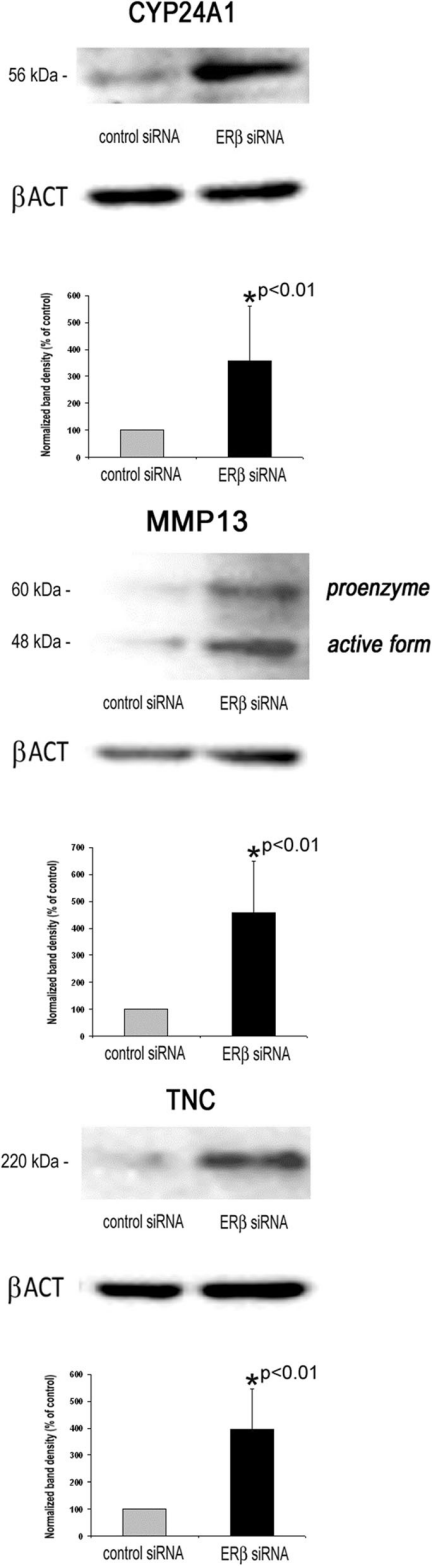
Western blot analysis of genes exhibiting elevated transcript levels after ERβ knockdown in MDA-MB-231 cells. Analyses were performed using specific antibodies against MMP13, TNC and CYP24A1 and β-actin as a loading control. Shown are representative blots and the means of three experiments (*n* = 3). *vs. control siRNA (unpaired t‑test, two‑tailed)

Transcript levels of 55 genes were found to be induced more than 2-fold, whereas 16 genes were more than 2-fold decreased in MDA-MB-231 cells transfected with ERβ siRNA (Table 1). Additional Western Blot analyses were performed which were able to corroborate upregulation of CYP24A1, MMP13 and TNC on the protein level in ERβ knockdown cells (Fig. 3).

**Table 1:** Effect of an ERβ knockdown on transcriptome of MDA-MB-231 cells as assessed by means of Affymetrix Human Gene 1.0 ST arrays. Shown are all genes exhibiting more than 2-fold change with a *p*-value <0.05 (*n* = 2)

Fold change	*p*-value	Gene symbol	Gene name
Upregulated genes			
4,82	0,01331	CYP24A1	cytochrome P450, family 24, subfamily A, polypeptide 1
4,23	0,00240	CXCL14	chemokine (C-X-C motif) ligand 14
3,80	0,02136	ENC1	ectodermal-neural cortex 1 (with BTB-like domain)
3,71	0,01270	DKK1	dickkopf 1 homolog (Xenopus laevis)
3,38	0,00778	HIPK3	homeodomain interacting protein kinase 3
3,36	0,02378	TRIM49B	tripartite motif containing 49B
3,21	0,02620	MMP13	matrix metallopeptidase 13 (collagenase 3)
3,04	0,00236	ARRDC3	arrestin domain containing 3
2,98	0,00932	TNC	tenascin C
2,82	0,04833	KRT4	keratin 4
2,82	0,02350	FRG2B	FSHD region gene 2 family, member B
2,74	0,04198	LOC100506966	uncharacterized LOC100506966
2,72	0,00962	AQP1	aquaporin 1 (Colton blood group)
2,62	0,01339	MFAP5	microfibrillar associated protein 5
2,61	0,03991	ANGPT1	angiopoietin 1
2,50	0,02567	MAPK8IP1	mitogen-activated protein kinase 8 interacting protein 1
2,49	0,00499	DEPTOR	DEP domain containing MTOR-interacting protein
2,48	0,04712	IGFBP5	insulin-like growth factor binding protein 5
2,43	0,01205	NPNT	nephronectin
2,41	0,01544	C12orf53	chromosome 12 open reading frame 53
2,37	0,03051	HSPA2	heat shock 70 kDa protein 2
2,36	0,02207	PLLP	plasmolipin
2,36	0,02959	SLC47A2	solute carrier family 47, member 2
2,35	0,01648	CRIP1	cysteine-rich protein 1 (intestinal)
2,33	0,00857	CNIH2	cornichon homolog 2 (Drosophila)
2,30	0,00344	TGFB2	transforming growth factor, beta 2
2,27	0,02260	CACNG4	calcium channel, voltage-dependent, gamma subunit 4
2,27	0,02987	CYP4F2	cytochrome P450, family 4, subfamily F, polypeptide 2
2,26	0,02720	MTRNR2L2	MT-RNR2-like 2
2,25	0,01804	TRIM53AP	tripartite motif containing 53A, pseudogene
2,20	0,00819	TP53INP1	tumor protein p53 inducible nuclear protein 1
2,19	0,02351	CLEC2L	C-type lectin domain family 2, member L
2,19	0,00476	DRAM1	DNA-damage regulated autophagy modulator 1
2,19	0,00903	TRIM49L1	tripartite motif containing 49-like 1
2,19	0,00903	TRIM49L1	tripartite motif containing 49-like 1
2,16	0,02128	PTGER4	prostaglandin E receptor 4 (subtype EP4)
2,13	0,01616	FLRT3	fibronectin leucine rich transmembrane protein 3
2,12	0,02280	DPYSL2	dihydropyrimidinase-like 2
2,12	0,03376	ATP6V1B1	ATPase, H+ transporting, lysosomal 56/58 kDa, V1 subunit B1
2,10	0,04897	FAM102B	family with sequence similarity 102, member B
2,10	0,00007	CHST15	carbohydrate (N-acetylgalactosamine 4-sulfate 6-O) sulfotransferase 15
2,10	0,00777	PTBP3	polypyrimidine tract binding protein 3
2,09	0,02408	TGFB1	transforming growth factor, beta 1
2,07	0,04539	NID1	nidogen 1
2,07	0,03066	IGFBP7	insulin-like growth factor binding protein 7
2,07	0,00551	LOC100509553	ETS domain-containing protein Elk-1-like
2,05	0,03765	PPP1R3C	protein phosphatase 1, regulatory subunit 3C
2,04	0,00619	FOXN1	forkhead box N1
2,03	0,01322	GPR56	G protein-coupled receptor 56
2,03	0,01062	SFN	stratifin
2,02	0,00706	CYTL1	cytokine-like 1
2,02	0,00298	PRICKLE1	prickle homolog 1 (Drosophila)
2,01	0,00401	MET	met proto-oncogene (hepatocyte growth factor receptor)
2,00	0,02964	MLLT11	myeloid/lymphoid or mixed-lineage leukemia (trithorax homolog, Drosophila); translocated to, 11
2,00	0,01646	LOX	lysyl oxidase
Downregulated genes			
−2,00	0,01776	GK5	glycerol kinase 5 (putative)
−2,01	0,00969	KPNA5	karyopherin alpha 5 (importin alpha 6)
−2,04	0,02721	TMC7	transmembrane channel-like 7
−2,05	0,04973	C4orf27	chromosome 4 open reading frame 27
−2,06	0,01484	IARS	isoleucyl-tRNA synthetase
−2,08	0,00684	TAF9B	TAF9B RNA polymerase II, TATA box binding protein (TBP)-associated factor, 31 kDa
−2,11	0,03884	JHDM1D	jumonji C domain containing histone demethylase 1 homolog D (S. cerevisiae)
−2,13	0,01199	ODC1	ornithine decarboxylase 1
−2,19	0,03799	MCMDC2	minichromosome maintenance domain containing 2
−2,22	0,04674	GPD2	glycerol-3-phosphate dehydrogenase 2 (mitochondrial)
−2,23	0,01299	MIR320D2	microRNA 320d-2
−2,24	0,00323	DICER1	dicer 1, ribonuclease type III
−2,29	0,02510	RN5S505	RNA, 5S ribosomal 505
−2,38	0,02642	LINC00243	long intergenic non-protein coding RNA 243
−2,55	0,04468	MTX3	metaxin 3
−2,62	0,00555	SEMA3D	sema domain, immunoglobulin domain (Ig), short basic domain, secreted, (semaphorin) 3D

**Fig. 4:**
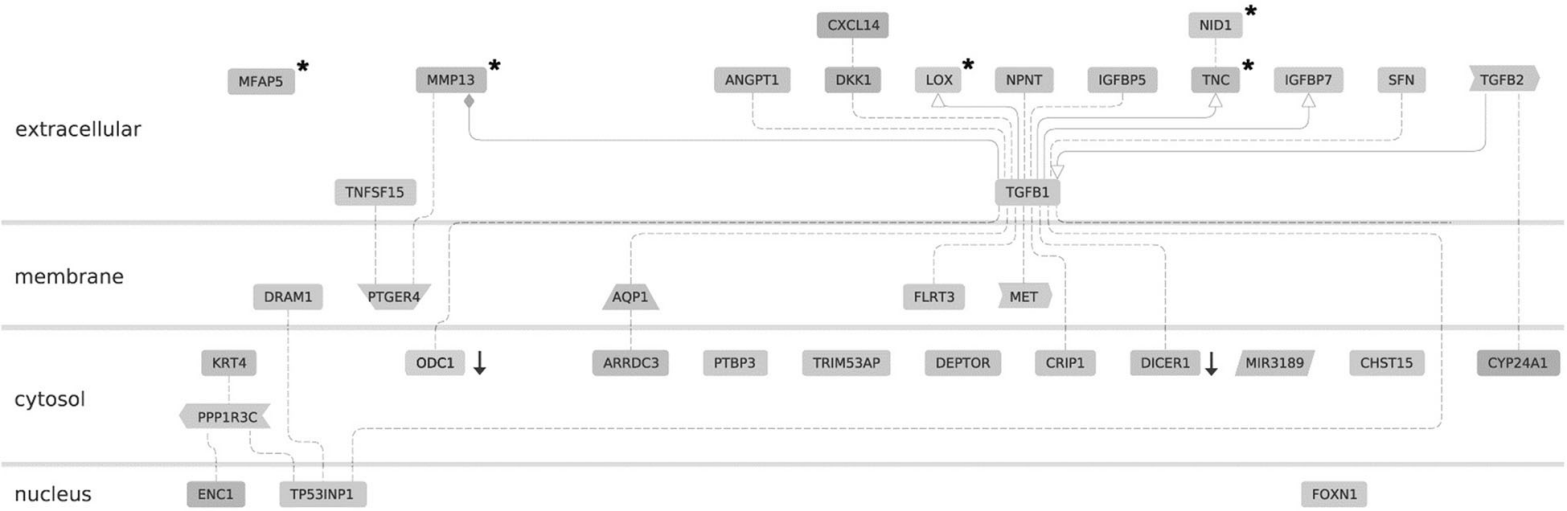
Network of genes regulated after ERβ knockdown in MDA-MB-231 cells and cellular location of their gene products. In DNA microarray analyses, the indicated genes were found to be induced more than 2-fold (*p* < 0.05) after ERβ knockdown, except the two genes marked with a *black arrow*, which were down regulated at least 2-fold. * = protein with function in extracellular matrix. *Dashed line*: association of undefined type due to co-citation of gene A and B; *solid line with arrow*: activation of gene B by gene A; *solid line with rhombus*: modulation of gene B by gene A. This figure was created using Genomatix Pathway Analysis software (Genomatix, Munich, Germany)

Microarray data were then processed using Genomatix Software Suite (Genomatix Software, Munich, Germany) and Ingenuity Pathway Analysis software (IPA, Ingenuity Systems, USA). With regard to localization in cellular components, Genomatix software revealed that as much as 20 of the upregulated genes were associated with the Gene Ontology (GO) term *extracellular region,* among them five genes coding for extracellular matrix proteins and ten genes with known functions in the extracellular matrix (Table 2). Analysis of the data with regard to the GO domain *molecular function* revealed that beside proteins TGFB1 and B2 being ligands for transforming growth factor receptors, other regulated genes coded for proteins being able to bind to fibronectin, lipoprotein particle receptors, insulin-like growth factor receptors or cytokine receptors. With regard to biological processes, the regulated genes were found to be associated with assembly or organization of the extracellular matrix, but also with tissue morphogenesis, apoptosis, cell adhesion and migration. A set of genes was known to be regulated in response to steroid hormones like estrogens (Table 2). By means of Genomatix Pathway Analysis software, the genes regulated by ERβ knockdown in MDA-MB-231 cells could be connected by a network of genes known to be regulated by TGFB1 (Fig. 4).

**Table 2:** Genes with more than 2-fold regulation after knockdown of ERβ: Gene ontology (GO)

GO-Term	GO-Term id	*P*-value	List of observed genes
A. GO Cellular components			
Extracellular matrix part	GO:0044420	4,91E-04	MFAP5, TNC, MMP13, LOX, NID1
Extracellular region	GO:0005576	3,71E-05	MFAP5, AQP1, TNC, IGFBP7, NPNT, MMP13, LOX, CXCL14, TNFSF15, ANGPT1, TGFB2, MTRNR2L2, FLRT3, SEMA3D, TGFB1, NID1, DKK1, CYTL1, IGFBP5, SFN
Extracellular matrix	GO:0031012	2,66E-06	MFAP5, TNC, IGFBP7, NPNT, MMP13, LOX, TGFB2, FLRT3, TGFB1, NID1
B. GO Molecular functions			
Type II transforming growth factor beta receptor binding	GO:0005114	2,73E-04	TGFB2, TGFB1
Fibronectin binding	GO:0001968	2,16E-03	MMP13, IGFBP5
Lipoprotein particle receptor binding	GO:0070325	2,16E-03	MMP13, DKK1
Insulin-like growth factor binding	GO:0005520	3,74E-03	IGFBP7, IGFBP5
Cytokine receptor binding	GO:0005126	6,89E-03	CXCL14, TNFSF15, TGFB2, TGFB1
C. GO Biological processes (excerpt)			
Extracellular matrix assembly	GO:0085029	4,84E-04	LOX, TGFB1
Tissue morphogenesis	GO:0048729	6,67E-04	TNC, TGFB2, TGFB1, PRICKLE1, DKK1, IGFBP5, DICER1
Regulation of apoptotic process	GO:0042981	9,83E-04	AQP1, MLLT11, DEPTOR, TAF9B, ANGPT1, TP53INP1, TGFB2, TGFB1, MAPK8IP1, DICER1, HIPK3, SFN
Extracellular matrix organization	GO:0030198	1,43E-03	LOX, TGFB2, TGFB1, NID1
Response to steroid hormone stimulus	GO:0048545	5,18E-03	AQP1, MMP13, LOX, TGFB2, TGFB1
Cell adhesion	GO:0007155	6,37E-03	TNC, IGFBP7, NPNT, ANGPT1, GPR56, TGFB2, FLRT3, TGFB1, NID1
Regulation of cell migration	GO:0030334	7,78E-03	PTGER4, ANGPT1, TGFB2, TGFB1, IGFBP5

**Fig. 5:**
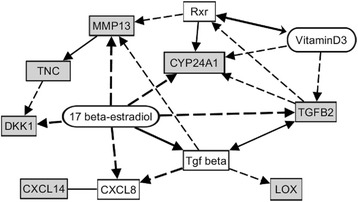
Network of genes induced after ERβ knockdown including the known effects of 17 β-estradiol on their expression. *Grey boxes*: up-regulated genes. *Dashed line*: regulation of expression, *solid line:* (additionally) other forms of interactions. The figure was created by means of Ingenuity Pathway Analysis software (Ingenuity Systems, Redwood City, USA)

Further analyzes of the microarray data by means of Ingenuity Pathway Analysis software (IPA, Ingenuity Systems) generated a second gene network including involvement of estrogen signaling (Fig. 5).

### Role of CYP24A1 and CXCL14 in invasion of MDA-MB-231 breast cancer cells

**Fig. 6:**
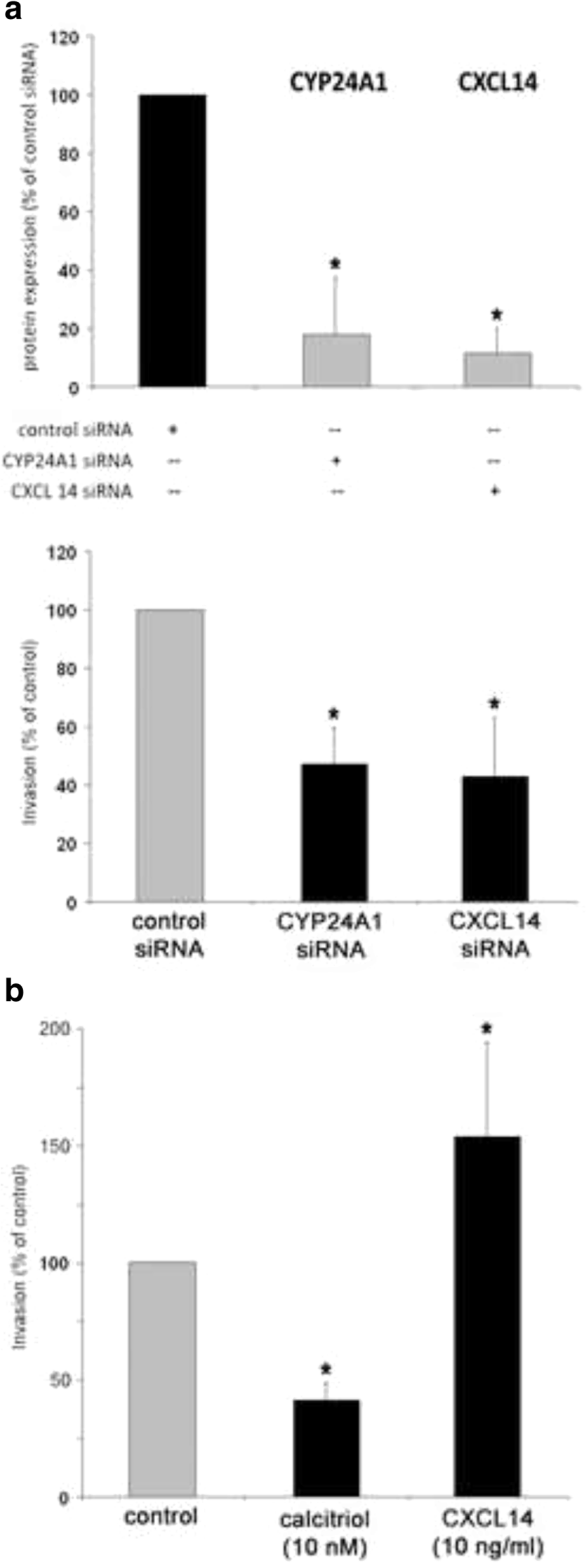
Effect of CYP24A1, 1,25(OH)_2_D_3_ (calcitriol) and CXCL14 on invasion of MDA-MB-231 cells. **a** Upper panel: Densitometrical analysis of Western blot experiments examining protein expression of the indicated genes 72 h after treatment with siRNA to CYP24A1 or CXCL14. Values are expressed in percentage of protein expression in control-transfected cells (*n* = 3). Lower panel: Invasion of MDA-MB-231 cells determined 72 h after treatment with siRNA to CYP24A1 or CXCL14, in percentage of invasion of control-transfected cells (*n* = 3). **b** Untransfected cells were treated with calcitriol (10 nM) or recombinant CXCL14 (10 ng/ml) 48 h before and during invasion assay. Cellular invasion was examined in vitro like described in the Materials and Methods section. Values are expressed in percentage of invasion of vehicle-treated cells (*n* = 3). *p < 0.01 vs. control (unpaired t‑test, two‑tailed)

Given that CYP24A1 and CXCL14 were the top upregulated genes, we further examined their role in MDA-MB-231 breast cancer cell invasion. For this purpose, we knocked down their expression by means of siRNA transfection and examined the effect on invasion of MDA-MB-231 cells. Three days after siRNA transfection, specific protein levels were reduced by 89.4% (CXCL14, *p* < 0.01), or 82.1% (CYP24A1, *p* < 0.01), respectively (Fig. 6a **,** upper panel). Three days after siRNA transfection, knockdown of CYP24A1 gene resulted in significant inhibition of invasiveness down to 45.6% (*p* < 0.01), and knockdown of CXCL14 expression decreased MDA-MB-231 cell invasion down to 41.0% (Fig. 6a, lower panel).

To corroborate the data generated by knockdown of CYP24A1 and CXCL14, we treated this cell line with calcitriol or CXCL14 chemokine the day before and during the invasion assay. Calcitriol (10 nM) reduced invasion of MDA-MB-231 breast cancer cells by 59.8%, and CXCL14 (10 ng/ml) increased their invasiveness up to 151.9% (Fig. 6b).

## Discussion

In this study, invasiveness of TNBC cells in vitro was found to decrease after treatment with ERβ agonists, but increased after knockdown of this gene. The results of our study clearly suggest that ERβ might be able to suppress invasion of certain breast cancer cells in an ERα-independent manner.

Although the role of ERβ in breast cancer is only beginning to be understood, an increasing amount of evidence suggests that this receptor might exert tumor-suppressor functions [13–17]. If this is the case, activation of ERβ by specific agonists might be a feasible treatment option for breast cancer. In this study, we tested four ERβ agonists which have been reported to bind preferentially to this receptor, but only to a much smaller extent to ERα. Given that the employed cell lines were ERα-negative, the observed agonist effects most likely were mediated by ERβ.

The dihydrotestosterone metabolite 5α-androstane-3β, 17β-diol (3β-Adiol) is an androgen derivative which does not bind androgen receptors, but efficiently binds ERβ, and its serum concentration is known to decline in the second half of life [22]. 3β-Adiol acts as a physiological ERβ-activator in different tissues [23, 24]. In line with our data, 3β-Adiol has been reported to inhibit cellular migration and epithelial-mesenchymal transition of prostate cancer cells as well as to reduce tumor progression [22, 25]. ERB-041 and WAY-200070 are highly specific synthetic ERβ agonists [26, 27]. ERB-041 is known to display a more than 200-fold selectivity for ERβ than for ERα (EC_50_ ERβ = 2 nM), WAY-200070 still has a 68-fold higher selectivity for ERβ than for ERα (EC_50_ ERβ = 2 nM) [28]. Liquiritigenin is a plant-derived flavonoid from licorice root, which acts as a highly selective agonist of ERβ (EC_50_ ERβ = 36.5 nM) [29]. Previously, Liquiritigenin and 3β-Adiol have been reported to inhibit proliferation of different breast cancer cell lines except of TNBC cell line MDA-MB-231, while the agonists WAY200070 and ERB-041 did not affect proliferation of ERα-positive breast cancer cells lines [30, 31].

The fact that invasiveness of HS578T cells was only sensitive to high ERβ agonist concentrations might be explained by the significantly lower ERβ expression levels observed in this cell line. Our observation that only lower concentrations of the ERβ agonists were able to reduce invasion of MDA-MB-231 cells is in line with the agonists EC_50_ values for ERβ, which all are in the low nanomolar range. Pathway analyses of the microarray data revealed induction of several genes coding for components or regulators of the extracellular matrix after knockdown of ERβ in MDA-MB-231 cells. Many genes regulated after treatment with ERβ siRNA could be identified to exert important functions in cell adhesion, cell migration and tumor cell invasion. Most of these genes are known targets of TGFβ like MMP13, TNC, IGFBP7 and LOX [32–34]. Due to the assessed transcriptome changes and upregulation of TGFB1 and TGFB2 itself, it is suggested that knockdown of ERβ resulted in activation of TGFβ1/2 signaling, which resulted in induction of genes with known functions in extracellular matrix and tumor cell invasion (Fig. 5). TGFβ signaling previously has been shown to induce motility and invasion of breast cancer cells [35–40].

TNC, whose expression was shown to be significantly induced after knockdown of ERβ both on the mRNA and protein level, is known to exert important functions in tumor cell invasion. TNC gene codes for the large extracellular matrix glycoprotein tenascin-c that shows prominent stromal expression in many solid tumors and has been reported to promote invasion of tumor cells of different origin, including breast cancer cells by matrix metalloproteinase-dependent and -independent mechanisms [41–47].

MMP13 gene, the expression of which was induced more than 3-fold after ERβ knockdown, codes for matrix metalloproteinase 13 (collagenase 3), a protease originally identified in breast carcinoma, which is also known to promote tumor cell invasion and has been previously reported as a molecular marker for transition of ductal carcinoma in situ lesions to invasive ductal carcinomas [48].

The observed activation of CYP24A1 gene coding for a mitochondrial enzyme which degrades 1,25-dihydroxyvitamin D_3_ into biologically inactive metabolites is another key event triggered by ESR2 knockdown. The complementary effects of CYP24A1 gene knockdown and treatment with calcitriol on invasion we observed corroborate the proposed key role of this gene in our study. CYP24A1 antagonizes the antitumoral actions of this vitamin and is known to be overexpressed in breast cancer tissue [49, 50]. The observed upregulation of CYP24A1 mRNA and protein after knockdown of ERβ is proposed to be one important reason for the increase of MDA-MB-231 cell invasiveness, because 1,25-dihydroxyvitamin D_3_ is known to reduce invasion, motility and metastasis of cancer cells (reviewed in [51]). Though we did not measure the exact concentration of 1,25-dihydroxyvitamin D_3_ in the fetal calf serum we used for cell culture, it is well known that serum contains calcitriol. Given that this vitamin is known to suppress expression of tenascin-c, the upregulation of this ECM component we found both on the mRNA and protein level most probably is the consequence of the elevated CYP24A1 expression we observed [52].

The strongly induced chemokine CXCL14 is known to exert both tumor-suppressive and tumor-promoting effects in mammals. While it primarily acts as a chemo-attractor for macrophages, dendritic cells and natural killer cells, CXCL14 is also able to act as a pro-tumorigenic factor. In line with our findings, CXCL14 has been previously reported to promote motility and invasion of breast and prostate cancer cells and bone metastasis of lung cancer cells [53–55]. CXCL14 has previously been reported to be induced by steroid hormones like progestin, but the mechanism linking ERβ and CXCL14 remains to be elucidated [56].

Pathway analyzes finally generated a gene network which might be able to at least partially elucidate the connection between ERβ knockdown and the transcriptome changes we observed (Fig. 6). Expression of the genes CYP24A1 and MMP13 has previously been reported to be activated by estrogens [57, 58]. Thus, it is tempting to speculate that repression of ERβ - knowing to act as an ERα antagonist in certain settings - might increase estrogen-triggered expression of MMP13 and CYP24A1 mediated by ERα. Expression of TGFβ, a key molecule of the generated network is also known to be regulated by estrogens, but this interaction seems to be more complex. Whereas some studies reported activation of TGFβ expression by estrogens, others found inhibitory effects of this steroid hormone particularly on expression of TGFB2 [59, 60]. Thus, the molecular mechanisms underlying the observed activation of TGFβ-expression and -signaling triggered by knockdown of ESR2 gene remain to be elucidated.

## Conclusion

In conclusion, the observed effects of an ERβ knockdown and of treatment with ERβ agonists on breast cancer cell invasion were consistent and clearly suggest that this receptor inhibited invasion of the employed TNBC cells in vitro. Transcriptome and gene network analyses provided molecular mechanisms which might underlie the observed alteration of invasion. Whether ERβ agonists might be suitable for treatment of triple-negative breast cancer, has to be evaluated in further animal and clinical studies.

## Additional files

Additional file 1: File S1 Primers used for RT-qPCR. (PDF 14 kb)
Additional file 2: Figure S2. Receptor expression of MDA-MB-231 and HS578T breast cancer cells. Expression of the indicated receptors was assessed by means of RT-qPCR and is shown on the mRNA level in percentage of maximum expression in MCF-7 (or SK-BR-3) cells (*n* = 3). (JPG 94.5 kb)
Additional file 3: Figure S3. Relative invasion capacity of MDA-MB-231 and HS578T cells. In vitro invasion was assessed 48 h after seeding the indicated cell lines on top of a reconstituted basement membrane gel as described in the Materials and Methods section. Invasion of MDA-MB-231 cells was set as 100% (*n* = 4). The difference in invasion capacity between both cell lines did not reach a statistically significant level (unpaired t‑test, two‑tailed). (JPG 44 kb)
Additional file 4: Figure S4. Proliferation of MDA-MB-231 and HS578T breast cancer cells after knockdown of ESR2 gene expression. The day after transfection with negative control siRNA or ESR2 siRNA, cells were seeded in DMEM/F12 plus 10% FSC in triplicates and relative numbers of viable cells were measured on day 0, 2, 3 and 4 using the Cell Titer Blue assay (Promega) as described in the Materials and Methods section. Values are expressed in percent of proliferation of mock-transfected cells (*n* = 3). **p* < 0.05 vs. mock-transfected cells (Kruskal-Wallis H-test with Bonferroni post-hoc test). (JPG 41 kb)

## Abbreviations

CK: Cytokeratin
DMEM: Dulbecco’s modified eagle’s medium
DNA: Deoxyribonucleic acid
ERβ: Estrogen receptor beta
FCS: Fetal calf serum
GO: Gene ontology
PCR: Polymerase chain reaction
qPCR: Quantitative polymerase chain reaction
RNA: Ribonucleic acid
RT: Reverse transcription
siRNA: Short interfering ribonucleic acid
TGFβ: Transforming growth factor beta
TNBC: Triple negative breast cancer
