# Supplementary material for: Agonists and knockdown of estrogen receptor β differentially affect invasion of triple-negative breast cancer cells in vitro
Source: BMC Cancer. 2016 Dec 21;16:951. doi: 10.1186/s12885-016-2973-y (PMC5178087; doi:10.1186/s12885-016-2973-y)
Supplement: Additional file 1: — File S1 Primers used for RT-qPCR. (PDF 14 kb) [file 12885_2016_2973_MOESM1_ESM.pdf]

Supplemental file S1

PCR primer sequences used for *real* RT-qPCR

Gene	Primer (5´-3´)
ERα	CACATGAGTAACAAAGGCATGG<br>ATGAAGTAGAGCCCGCAGTG
ERβ	GGCATGCGAGTAACAAGGGC<br>GGGAGCCCTCTTTGCTTTT
PR	CAACTACCTGAGGCCGGATT<br>CATTGCCCTCTTAAAGAAGACCT
HER2	CATGGTCAAATGTTGGATGATT<br>CCTCATTCTGGATGACCACA
EGFR	CGAGGGCAAATACAGCTT<br>AAATTCACCAATACCTATT
