# Supplementary figures and images for: Agonists and knockdown of estrogen receptor β differentially affect invasion of triple-negative breast cancer cells in vitro
Source: BMC Cancer. 2016 Dec 21;16:951. doi: 10.1186/s12885-016-2973-y (PMC5178087; doi:10.1186/s12885-016-2973-y)

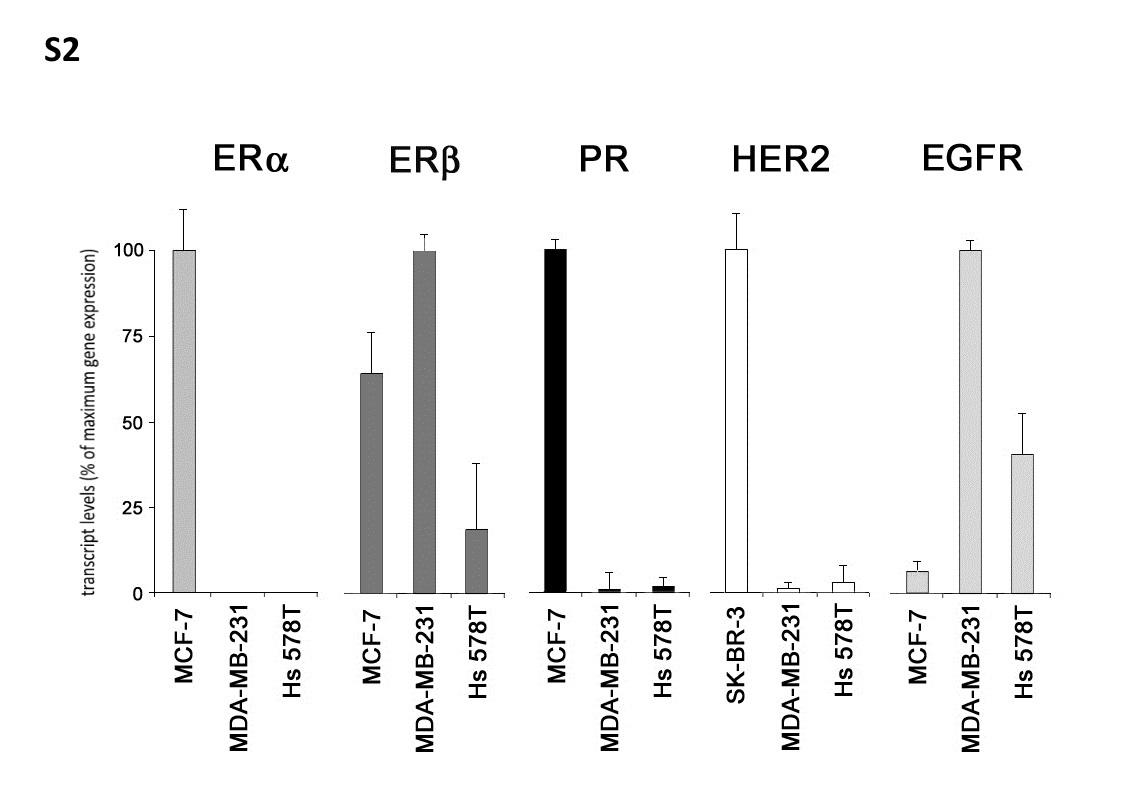

Supplement: Additional file 2: Figure S2. — Receptor expression of MDA-MB-231 and HS578T breast cancer cells. Expression of the indicated receptors was assessed by means of RT-qPCR and is shown on the mRNA level in percentage of maximum expression in MCF-7 (or SK-BR-3) cells (n = 3). (JPG 94.5 kb) [file 12885_2016_2973_MOESM2_ESM.jpg]

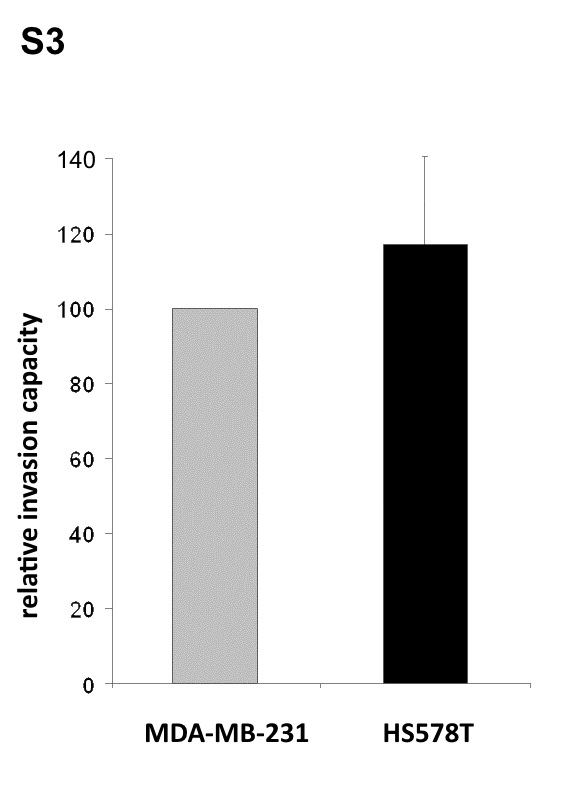

Supplement: Additional file 3: Figure S3. — Relative invasion capacity of MDA-MB-231 and HS578T cells. In vitro invasion was assessed 48 h after seeding the indicated cell lines on top of a reconstituted basement membrane gel as described in the Materials and Methods section. Invasion of MDA-MB-231 cells was set as 100% (n = 4). The difference in invasion capacity between both cell lines did not reach a statistically significant level (unpaired t‑test, two‑tailed). (JPG 44 kb) [file 12885_2016_2973_MOESM3_ESM.jpg]

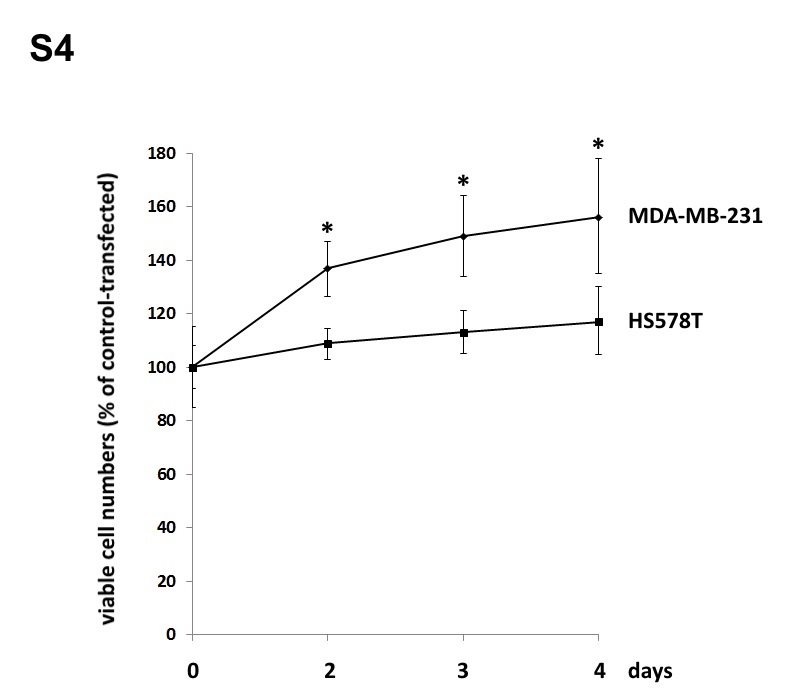

Supplement: Additional file 4: Figure S4. — Proliferation of MDA-MB-231 and HS578T breast cancer cells after knockdown of ESR2 gene expression. The day after transfection with negative control siRNA or ESR2 siRNA, cells were seeded in DMEM/F12 plus 10% FSC in triplicates and relative numbers of viable cells were measured on day 0, 2, 3 and 4 using the Cell Titer Blue assay (Promega) as described in the Materials and Methods section. Values are expressed in percent of proliferation of mock-transfected cells (n = 3). *p < 0.05 vs. mock-transfected cells (Kruskal-Wallis H-test with Bonferroni post-hoc test). (JPG 41 kb) [file 12885_2016_2973_MOESM4_ESM.jpg]
